# Balloon Cryoablation for the Treatment of Paroxysmal Atrial Fibrillation

**DOI:** 10.5935/abc.20170108

**Published:** 2017-09

**Authors:** Guilherme Fenelon, Frederico Scuotto, Claudio Fischer, Marco Antonio Perin, Marcia Makdisse, Angelo Amato Vincenzo de Paola

**Affiliations:** 1 Hospital Israelita Albert Einstein, São Paulo, SP - Brazil; 2 Universidade Federal de São Paulo (UNIFESP), São Paulo, SP - Brazil

**Keywords:** Atrial Fibrillation / therapy, Catheter Ablation / methods, Cryosurgery / methods, Cryosurgery / trends

## Introduction

Radiofrequency ablation (RFA) aiming at the electrical isolation of the pulmonary vein (PV) is more effective in controlling the rhythm than drugs, especially in young individuals with symptomatic paroxysmal atrial fibrillation (AF) without structural heart disease.^1-3^ Electrical isolation is performed with point-by-point RFA around the PV, constituting a complex and lengthy procedure.^2,3^ Recently, technologies have been developed aiming to simplify PV isolation, among them the balloon cryoablation.^2,4^ In this approach, the cryoballoon is inflated in the PV ostium in order to completely occlude it. The release of cryoenergy cools the balloon surface, having the potential to isolate the vein with a single application. The efficacy and safety of cryoablation are similar to RFA, but the procedure is faster.^4-7^

Although used worldwide,^2-4^ only recently cryoablation was made available in Brazil. The objective of this study was to report the first three cases performed in our country using this technology.

## Case Reports

Patients were included in a study approved by the National Research Ethics Commission (Comissão Nacional de Ética em Pesquisa - CONEP), number 03094112.9.0000.0071, after signing the Free and Informed Consent form. All patients met the following inclusion criteria: (1) documented symptomatic paroxysmal AF; (2) at least two episodes in the last 3 months; (3) refractory to at least one antiarrhythmic drug.

### Case 1

Male patient, 36 years old, hypertension controlled with losartan, with paroxysmal AF for the last 5 years, had used sotalol without success. He remained symptomatic (palpitations) with the use of propafenone, atenolol and dabigatran (CHA2DS2-VASc score 1).^8^ The echocardiogram showed a slight increase in the left atrium (anteroposterior diameter of 44 mm) and normal ventricular function (left ventricular ejection fraction of 65%).

### Case 2

Female patient, 67 years old, with hypertension, dyslipidemia and hypothyroidism, controlled with enalapril, metoprolol, levothyroxine and rosuvastatin. She had paroxysmal AF crisis for the last 3 years and remained symptomatic (palpitations) while using propafenone and rivaroxaban (CHA2DS2-VASc score 3).^8^ She used amiodarone, withdrawn due to hypothyroidism. Echocardiography showed normal left atrium (anteroposterior diameter of 39 mm) and ventricular function (left ventricular ejection fraction of 74%).

### Case 3

Male patient, 50 years old, hypertension controlled with losartan and chronic bronchitis due to smoking, paroxysmal AF for the last 10 years, had used sotalol and amiodarone without success. He was symptomatic (palpitations) while using propafenone and dabigatran (CHA2DS2-VASc score 1).^8^ The echocardiogram showed a slight increase in the left atrium (anteroposterior diameter of 44 mm), normal ventricular function (left ventricular ejection fraction of 63%) and mild mitral escape.

**Figure 1:**
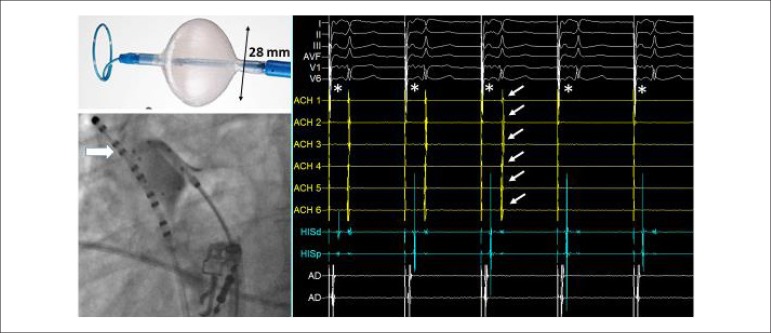
The upper left panel shows the 28-mm diameter cryoballoon and the octapolar circular catheter, used to measure real-time isolation of the pulmonary veins. In the lower left panel, fluoroscopic image showing the balloon catheter inflated in the ostium of the right superior pulmonary vein of the third case. Contrast retention inside the vein indicates vessel occlusion. Observe the circular catheter inside the vein and the decapolar catheter (arrow) placed in the upper right atrium for phrenic stimulation. In the right panel, intracavitary tracings showing the isolation of the same vein. From top to bottom, surface electrocardiogram (leads I, II, III, aVF, V1 and V6); electrograms recorded inside the vein by the circular catheter (ACH 1, 2 ACH, ACH 3, 4 ACH, ACH 5 and ACH 6); atrial and ventricular electrograms collected by the catheter positioned in the bundle of His (His D and His P); and electrograms of the two pairs of distal electrodes of the decapolar catheter in upper right atrium. Observe the sudden disappearance of the pulmonary vein potentials (arrows) 40 seconds after application of cryoenergy, indicating electrical isolation. The asterisks indicate the spicules of the continuous phrenic nerve stimulation by the catheter in the upper right atrium at 60 bpm cycles. Observe that the stimulation also commands the atria. Tracings at 50 mm/second*.*

The procedures were performed on November 3, 2014, by the same team, with the help of an instructor, using standardized techniques.^9^ Antiarrhythmic and anticoagulant drug use was suspended five days before and on the day before the procedure, respectively. Imaging methods for anatomical definition of the PV were not used. Transesophageal echocardiography was performed under general anesthesia, to exclude thrombi. Then, femoral venous access was obtained for allocation of decapolar and quadruple catheters in the coronary sinus and right atrium. With the aid of transesophageal echocardiography, a single transseptal puncture was performed and an 8F sheath (SL1, St. Jude Medical) was placed in the left atrium, which subsequently was replaced by a 15F deflectable sheath (Flexcath, Medtronic), through which the second-generation, 28-mm diameter 10.5F balloon catheter (Arctic Front Advance, Medtronic) was introduced. Selective catheterization of the PV was performed with the Achieve octapolar circular catheter (Medtronic), which was also used to measure real-time electrical insulation. After cryoballoon insufflation and occlusion of each vein (measured by contrast luminal retention), two applications of cryoenergy were performed, lasting 3 minutes, aiming at a temperature of -40ºC in the catheter thermistor. The second application was a reinforcement one, for longer lasting of the lesion. To prevent injury to the phrenic nerve, applications in the right veins were performed under continuous phrenic stimulation through the decapolar catheter located in the upper right atrium (Figure 1). Esophageal temperature monitoring and full heparinization (ACT 300-350 seconds) were maintained.

**Figure 2:**
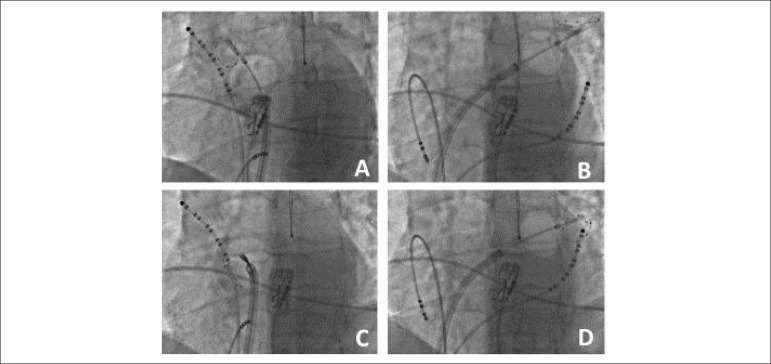
Fluoroscopic images in the left anterior oblique projection showing the position of the balloon catheter inflated in the ostium of the four pulmonary veins of the third case: (A) upper right; (B) upper left; (C) lower right; (D) lower left. In the B and C panels, observe the decapolar catheter positioned in the coronary sinus. This same catheter is subsequently used for phrenic stimulation in the upper right atrium during cryoablation of the right veins (A and D). Also, observe the esophageal thermometer (projecting on the spinal column).

Twelve PV were treated (Figure 2), and electrical isolation was achieved in all (100%), with a mean of 2.3 adequate applications (-40ºC) of cryoenergy per vein (mean of three applications per vein in the first patient and two per vein in the remaining). Applications with inadequate temperature were discontinued after approximately 30 seconds, and the balloon was repositioned. The mean procedure time (skin to skin) was 125 minutes (150, 150 and 75 minutes) and of fluoroscopy, 47 minutes (63, 47 and 33 minutes). The esophageal temperature did not change during the applications. At the end of the procedure, heparin was neutralized with protamine, hemostasis was obtained and a compressive dressing was performed. After recovery from anesthesia, the patients were sent to the infirmary, receiving enoxaparin (half the full dose - 0.5 mg/kg every 12 hours) during the first 24 hours. There were no complications, and all patients were discharged in the following morning, receiving the usual antiarrhythmic and anticoagulation drugs, in addition to omeprazole (for 30 days). The exams at hospital discharge (electrocardiogram and chest X-ray) were normal.

After a follow-up of 14 months, all patients remained asymptomatic and in sinus rhythm without antiarrhythmic drugs (withdrawn after 12 months), but receiving anticoagulants. There were no AF recurrences and the 24-hour Holter monitoring at 3, 6 and 12 months showed no arrhythmias.

## Discussion

These were the first cases in the country that used the cryoballoon for treatment of paroxysmal AF. It was possible to safely isolate the PV, attaining adequate control of the arrhythmia in the 14-month follow-up. These observations support the literature.^4-6^ In a multicenter randomized controlled trial comparing cryoablation with drugs, 70% (114/163) of the cryoablation group showed no recurrence after 1 year, compared with 7.3% (6/82) of the medication group. A systematic review reported 98% of immediate success with PV isolation, with maintenance of sinus rhythm of 72% after 1 year.^4^ Considering these results, cryoablation and point-by-point RFA were classified as standard techniques for ablation of paroxysmal AF.^2^

Studies comparing RF ablation with cryoballoon indicated success and similar complications, but cryoablation is faster.^4,6,7^ In this regard, one of our procedures was completed (skin to skin) in 75 minutes. While the overall rate of complications is similar, RFA tends to have a higher incidence of esophageal lesions and PV stenosis.^4^ This is because, compared to the heat lesion in RFA, cryothermic cooling lesions show less tissue breakdown, are more homogeneous and less thrombogenic.^4^ In contrast, cryoablation causes more phrenic lesions. Therefore, it is necessary to stimulate the phrenic nerve during the isolation of the right veins, immediately interrupting the application if there is any reduction in diaphragm contractions (Figure 1). With these measures, the incidence of permanent phrenic lesion is less than 0.3%.^4,7,9^ We had no complications in our series. It is noteworthy the fact that a randomized controlled trial was recently published, demonstrating the non-inferiority of cryoablation in relation to RFA regarding safety and efficacy.^10^

Cryoablation has a faster learning curve than the RFA.^4,5^ However, it is currently indicated only in paroxysmal AF, as the cryoballoon lesions are restricted to the PV antrum.^4,9^ The formation of linear lesions or the approach of other atrial regions require RF ablation.^1-3^

A limitation of this report is the sample size, which was small and obtained during the learning curve. The follow-up without antiarrhythmic drugs (two months) was short, but all patients were very symptomatic with frequent crises, despite the medication. The cost-effectiveness of the technique was not assessed.

This initial experience suggests that balloon cryoablation is effective and safe for fast PV isolation in patients with paroxysmal AF.
